# Book Reviews

**Published:** 1833-06

**Authors:** 


### BIBLIOGRAPHICAL NOTICES.

*A Manual of Pharmacy*. By Wm. Thomas Brande, f.r.s. &c. *Third Edition, corrected and enlarged.* London: Renshaw and Rush.

To every medical practitioner this work must be extremely convenient, as a book of ready reference upon all pharmaceutical subjects; and we have no better authority than Mr. Brande to whose guidance the student, who has yet to acquire the elementary parts of his profession, can intrust himself. It is divided into two parts; the first comprising the articles of the materia medica, the Latin names of which are arranged in alphabetical order; the second contains the pharmaceutical preparations and compounds, as directed by the London Pharmacopœia.

---

*The Black Death in the Fourteenth Century.* From the German of J. F. C. Hecker, m.d. Translated by B. G. Babington, m.d.—12mo. pp. 205. London: Schloss.

This is a very interesting little volume. Many of the facts which the author has selected respecting the dreadful pestilence described are important and curious. Our modern historians have but slightly alluded to the visitation of this most formidable disease, although in England it raged with frightful and fatal violence.

The chief motive which induced Dr. Babington to give us a translation of Hecker's original, was a desire that the study of the causes which have produced and propagated general pestilences, and of the moral effects by which they have been propagated, the most enlarged views should be taken. Here the contagionist and non-contagionist may each find ample support for his belief in particular cases; but, in the construction of a theory sufficiently comprehensive to explain throughout the origin and dissemination of universal disease, we shall not only perceive, Dr. Babington very truly observes, the insufficiency of either doctrine, taken singly, but, after admitting the combined influence of both, shall we then find our views too narrow, and be compelled, in our endeavours to explain the facts, to acknowledge the existence of unknown powers, wholly unconnected either with communication by contact or atmospheric contamination. The perusal of this little work will instruct the medical student, and amuse the general reader.

